# Supplementary material for: Patient-centred outcomes following non-operative treatment or appendicectomy for uncomplicated acute appendicitis in children
Source: BMJ Paediatr Open. 2023 Feb 6;7(1):e001673. doi: 10.1136/bmjpo-2022-001673 (PMC9906255; doi:10.1136/bmjpo-2022-001673)
Supplement: Author's manuscript [file bmjpo-2022-001673.draft_revisions.pdf]

BMJ Paediatrics Open

BMJ Paediatrics Open is committed to open peer review. As part of this commitment we make the peer review history of every article we publish publicly available.

When an article is published we post the peer reviewers' comments and the authors' responses online. We also post the versions of the paper that were used during peer review. These are the versions that the peer review comments apply to.

The versions of the paper that follow are the versions that were submitted during the peer review process. They are not the versions of record or the final published versions. They should not be cited or distributed as the published version of this manuscript.

BMJ Paediatrics Open is an open access journal and the full, final, typeset and author-corrected version of record of the manuscript is available on our site with no access controls, subscription charges or pay-per-view fees (http://bmjpaedsopen.bmj.com).

If you have any questions on BMJ Paediatrics Open's open peer review process please email info.bmjpo@bmj.com

BMJ Paediatrics Open

Patient centred outcomes following non-operative treatment or appendicectomy for uncomplicated acute appendicitis in children – results of a feasibility randomised controlled trial.

Journal:	*BMJ Paediatrics Open*
Manuscript ID	bmjpo-2022-001673
Article Type:	Original research
Date Submitted by the Author:	13-Sep-2022

Complete List of Authors:	Hall, Nigel; University of Southampton, University Surgery Unit, Faculty of Medicine; Southampton Children's Hospital, Department of Paediatric Surgery and Urology<br>Sherratt, Frances; University of Liverpool, Department of Health Services Research, Institute of Population Health Sciences<br>Eaton, Simon; UCL Great Ormond Street Institute of Child Health Population Policy and Practice<br>Walker, Erin; Great Ormond Street Hospital For Children NHS Trust<br>Chorozoglou, Maria; University of Southampton Faculty of Medicine, Southampton Health Technology Assessment Centre<br>Beasant, Lucy; University of Bristol School of Social and Community Medicine, Centre for Child and Adolescent Health<br>Stanton, Michael; Southampton Children's Hospital, Department of Paediatric Surgery and Urology<br>Corbett, Harriet; Alder Hey Children's NHS Foundation Trust, Department of Surgery<br>Rex, Dean; St George's University Hospitals NHS Foundation Trust, Paediatric Surgery<br>Hutchings , Natalie; University of Southampton, Southampton Clinical Trials Unit, Faculty of Medicine<br>Dixon, Elizabeth; University of Southampton, Clinical Trials Unit<br>Crawley, Esther; University of Bristol School of Social and Community Medicine, Centre for Child and Adolescent Health<br>Blazeby, Jane; University of Bristol School of Social and Community Medicine, Centre for Surgical Research<br>Young, Bridget; University of Liverpool, Department of Health Services Research<br>Reading, Isabel ; University of Southampton , Primary Care and Population Sciences, Faculty of Medicine

Keywords:	Gastroenterology

SCHOLARONE™
Manuscripts

I, the Submitting Author has the right to grant and does grant on behalf of all authors of the Work (as defined in the below author licence), an exclusive licence and/or a non-exclusive licence for contributions from authors who are: i) UK Crown employees; ii) where BMJ has agreed a CC-BY licence shall apply, and/or iii) in accordance with the terms applicable for US Federal Government officers or employees acting as part of their official duties; on a worldwide, perpetual, irrevocable, royalty-free basis to BMJ Publishing Group Ltd ("BMJ") its licensees and where the relevant Journal is co-owned by BMJ to the co-owners of the Journal, to publish the Work in this journal and any other BMJ products and to exploit all rights, as set out in our licence.

The Submitting Author accepts and understands that any supply made under these terms is made by BMJ to the Submitting Author unless you are acting as an employee on behalf of your employer or a postgraduate student of an affiliated institution which is paying any applicable article publishing charge ("APC") for Open Access articles. Where the Submitting Author wishes to make the Work available on an Open Access basis (and intends to pay the relevant APC), the terms of reuse of such Open Access shall be governed by a Creative Commons licence – details of these licences and which Creative Commons licence will apply to this Work are set out in our licence referred to above.

Other than as permitted in any relevant BMJ Author's Self Archiving Policies, I confirm this Work has not been accepted for publication elsewhere, is not being considered for publication elsewhere and does not duplicate material already published. I confirm all authors consent to publication of this Work and authorise the granting of this licence.

Confidential: For Review Only

Patient centred outcomes following non-operative treatment or appendicectomy for uncomplicated acute appendicitis in children – results of a feasibility randomised controlled trial.

Nigel J Hall[1*], Frances C Sherratt[2], Simon Eaton[3], Erin Walker[4], Maria Chorozoglou[5], Lucy Beasant[6], Michael Stanton[7], Harriet J Corbett[8], Dean Rex[9], Natalie Hutchings[10], Elizabeth Dixon[10], Esther Crawley[6], Jane Blazeby[11], Bridget Young[2], Isabel Reading[12]

[1]Faculty of Medicine, University of Southampton, Southampton, UK
[2]Department of Health Services Research, Institute of Population Health Sciences, University of Liverpool, Liverpool UK
[3]UCL Great Ormond Street Institute of Child Health, London, UK
[4]Great Ormond Street Hospital for Children NHS Foundation Trust, London, UK
[5]Southampton Health Technology Assessment Centre, Faculty of Medicine, University of Southampton, Southampton, UK
[6]Centre for Child and Adolescent Health, School of Social and Community Medicine, University of Bristol
[7]Department of Paediatric Surgery and Urology, Southampton Children's Hospital, University Hospital Southampton NHS Foundation Trust, UK
[8]Department of Paediatric Surgery, Alder Hey Children's NHS foundation Trust, East Prescott Road, Liverpool
[9]Department of Paediatric Surgery, St George's Hospital NHS foundation Trust, Tooting, London
[10]Southampton Clinical Trials Unit, University of Southampton, Southampton, UK
[11]Centre for Surgical Research, and the NIHR Bristol and Weston Biomedical Research Centre, Population Health Sciences, Bristol Medical School, University of Bristol and University Hospitals Bristol NHS Foundation Trust
[12]Primary Care and Population Sciences, Faculty of Medicine, University of Southampton, Southampton, UK

*Corresponding author

The authors have no competing interests to declare

Confidential: For Review Only

Abstract

Background

Whilst non-operative treatment is known to be effective for the treatment of uncomplicated acute appendicitis in children, comparative randomised trial data reporting important outcomes compared to appendicectomy are lacking. We report patient centred outcomes from a randomised controlled feasibility trial that aimed to determine the feasibility of a future large trial testing the effectiveness and cost-effectiveness of a non-operative treatment pathway compared to appendicectomy for the treatment of uncomplicated acute appendicitis in children.

Methods

Children (aged 4-15 years) in 3 tertiary paediatric surgery centres with a clinical diagnosis of uncomplicated acute appendicitis were randomised to a non-operative treatment pathway (comprising broad-spectrum antibiotics and active observation) or appendicectomy. Follow-up was for 6 months. Primary outcome was recruitment rate. Secondary outcomes reported here were clinical (focussed on clinical course, complications and recurrent appendicitis) and patient centred measures of recovery.

Results

Of 57 children enrolled in the trial, 3 withdrew. Twenty-seven were allocated to appendicectomy, of whom 8 developed a complication (3 requiring hospital readmission) and 27 were allocated to non-operative treatment of whom 19 (70%) were discharged home without undergoing appendicectomy. Nine of these had a readmission, including for recurrent appendicitis in 7. Duration of initial hospital stay was similar between treatment groups. Compared to appendicectomy, children in the non-operative treatment arm reported higher short term quality of life scores, shorter duration of requiring analgesia, more rapid return to normal activities and shorter parental absence from work.

Conclusions

These preliminary data suggest there may be differences in recovery profile and quality of life between treatment arms that are important to measure in a larger RCT comparing the effectiveness and cost effectiveness of these two different treatments.

Trial registration ISRCTN15830435.

Confidential: For Review Only

Introduction

Appendicectomy by either open or laparoscopic route is the mainstay of treatment for children with acute uncomplicated appendicitis. However, there is currently great interest in the use of non-operative treatment. Not only are several groups worldwide in the midst of research programs evaluating non-operative treatment compared to appendicectomy[1-7] but non-operative treatment of appendicitis in children has rapidly entered the armamentarium of many surgeons globally during the SARS-CoV-2 worldwide pandemic.[8 9]

One of the potential attractions of non-operative treatment of appendicitis to children and families is the avoidance of surgery and general anaesthesia, the avoidance of complications related to these, and the potential for a rapid recovery in the absence of surgical trauma. With this in mind, it is evident that reporting of outcomes specifically related to post-operative recovery is essential.

To date, reports of non-operative treatment for children with appendicitis have tended to focus on reporting outcomes most relevant to surgeons such as surgical complications and other treatments required.[10-12] Whilst these are clearly also of importance to patients and families, outcomes that we know are important to these groups such as how long they will spend in hospital and how much time parents will be required to take off work have been typically under-reported or missing entirely. The aim of this report is to describe the clinical and patient centred outcomes achieved in in a feasibility randomised controlled trial comparing non-operative treatment with appendicectomy for children with uncomplicated acute appendicitis; the feasibility outcomes have been reported already[2].

Methods

We performed a feasibility RCT comparing appendicectomy and non-operative treatment in children with suspected uncomplicated acute appendicitis. The study was designed to determine the feasibility of performing a subsequent larger trial to formally compare the clinical and cost-effectiveness of these very different types of treatment. This feasibility trial was open to recruitment in three specialist paediatric surgery centres in England for a 12 month period beginning March 2017 was conducted in accordance with a predefined published protocol[1] that was developed in accordance with SPIRIT-C guidance[13] and was registered prior to commencement (ISRCTN 15830435). The trial formed part of a wider project (CONservative TReatment of Appendicitis in Children – a randomised controlled feasibility Trial [CONTRACT]) that was funded by the National Institute of Health Research[14] and given ethical approval by the Hampshire A Research Ethics Committee (ref 16/SC/0596). The trial was overseen by an independent Trial Steering Committee

Confidential: For Review Only

(TSC) and Data Monitoring and Safety Committee (DMSC) and is reported in accordance with the CONSORT 2010 statement (extension for feasibility trials).[15] The feasibility results of the trial have been reported elsewhere.[2]

Participants

Children age 4 – 15 years (>3 and <16 years), with a clinical and/or radiological diagnosis of uncomplicated acute appendicitis which prior to study commencement would be treated with appendicectomy, were eligible for inclusion. Exclusion criteria were clinical signs or radiological findings to suggest perforated appendicitis, presentation with appendix mass, previous episode of appendicitis or appendix mass treated non-operatively, major anaesthetic risk precluding allocation to the appendicectomy arm, a known antibiotic allergy preventing allocation to non-operative treatment arm, antibiotic treatment started at referring institution (defined as 2 or more doses administered), cystic fibrosis, a positive pregnancy test, or current treatment for malignancy. There was no requirement for diagnostic imaging in this study since imaging is not routinely used in the diagnostic evaluation of children suspected to have appendicitis in the UK[16 17] and we wished to replicate current diagnostic pathways and deliver a pragmatic trial as far as possible.

Participant identification, recruitment and randomisation

Eligible participants were identified by the clinical team at the time of diagnosis, provided with written information about the trial and shown a short video describing the trial. After written informed consent (and assent from children aged 12 years or over who wished to give it), participants were randomised to one of two treatment arms in a 1:1 ratio via an independent web-based system allowing concealment of allocation prior to group allocation. Minimisation was used to ensure balance between groups in the following factors that may affect diagnostic accuracy and outcome of treatment: (a) sex: male; female; (b) age: 4-8; 9-15; (c) duration of symptoms (onset of pain to recruitment into study): <48 hours; ⩾48 hours, and (d) recruiting centre. The online system allowed complete pre-randomisation concealment of treatment allocation and provided instant assignment to either the Non-Operative treatment or Appendicectomy arm. Blinding was not considered ethical or feasible, so treating staff, parents and participants were aware of treatment allocation.

Trial Interventions

Confidential: For Review Only

Non-operative treatment arm

Children randomised to non-operative treatment were treated according to a clinical pathway designed specifically for this trial: fluid resuscitation, a minimum of 24 hours broad spectrum Intravenous (IV) antibiotics (per local antimicrobial policy), a minimum of 12 hours nil by mouth (NBM) and regular clinical review to detect signs and symptoms of significant clinical deterioration (including, but not limited to, increasing fever, increasing tachycardia, and increasing tenderness). After the initial 12-hour period of NBM, oral intake was advanced as tolerated. Children successfully treated without an operation were converted to oral antibiotics (per local policy) once they are afebrile for 24 hours and tolerating oral intake.

Clinical reviews were completed at approximately 24 and 48 hours post randomisation. Any children who showed signs of significant clinical deterioration by 24 hours, or at any point during the trial, were treated with appendicectomy. Children who were considered stable or improving continued with non-operative treatment. At 48 hours, any child who had not shown clinical improvement underwent an appendicectomy. The decision to continue non-operative treatment at these time points or to recommend discontinuation of non-operative treatment and appendicectomy, was made by the treating consultant based on clinical judgement. All reasons for change in treatment were recorded in detail in order to guide a clinical pathway in a future trial.

Any child who received an appendicectomy for an incomplete response to non-operative treatment, followed a standardised post-operative treatment regime already in use at each institution and identical to that used in the appendicectomy arm. The reason for having an appendicectomy was recorded.

Children treated non-operatively received a total of 10 days antibiotics following randomisation unless decided otherwise by the treating clinician. Children who received non-operative treatment were not routinely offered interval appendicectomy, but were counselled about the risk of recurrence.

Appendicectomy treatment arm

Children randomised to the appendicectomy arm underwent either open or laparoscopic appendicectomy at the surgeon's discretion, performed by a suitably experienced trainee (as per routine current practice) or a consultant. Participants received intravenous (IV) antibiotics from the time of diagnosis and were treated post-operatively with IV antibiotics according to existing institutional protocols. The duration of antibiotic therapy was not standardised due to anticipated variation in intra-operative findings and in response to treatment. The type of antibiotics used was identical to those used in the non-operative treatment arm within each centre. Any child failing to

Confidential: For Review Only

respond to first line antibiotics was treated as clinically appropriate with a longer course of antibiotics or a change in antibiotic therapy with the choice of antibiotic determined by intra-operative swab or fluid culture. Post-operatively, children with uncomplicated acute appendicitis or a normal appendix did not routinely have a nasogastric tube, nor a urinary catheter. They received oral intake as tolerated after surgery.

Discharge assessment and follow-up

Criteria for discharge home were identical in both treatment arms: vital signs within normal limits for age, afebrile for ⩾ 24 h, tolerating light diet orally, adequate oral pain relief and able to mobilise. Parents of all participants were given an information sheet at discharge informing them of 'red flag' symptoms and advised to contact their local unit if they had concerns about their child's clinical progress. Following discharge, all participants were asked to complete a diary card for each of the first 14 days that recorded information about medication taken (antibiotics and analgesia), whether they had been able to return to normal activity or full activity that day and if their parent(s) had had to take time off work as a consequence of their appendicitis. Follow-up visits for all participants took place at 6 weeks and at 3 and 6 months following discharge, either face-to-face or if not possible by telephone.

Outcomes

The primary outcome was the recruitment rate. This and other feasibility outcomes have been reported elsewhere.[2] Secondary trial outcomes reported here are:

- safety and overall success of initial non-operative treatment
- complications of disease and treatment (measured during hospital stay and 6 month follow-up period)
- length of hospital stay
- profile of post-discharge recovery (time to return to normal activities, time to return to full activities and duration of parental absence from work)
- rate of recurrent appendicitis during 6 month follow-up period
- health related quality of life (using the CHU-9D tool).

We have also reported all outcomes from a recently developed core outcome set for uncomplicated acute appendicitis in children where available.[18]

https://mc.manuscriptcentral.com/bmjpo

Confidential: For Review Only

Data collection and analysis

Data were recorded by dedicated research nurses directly into an electronic, secure, online case report form. Data analysis was performed by the study statistician who was blinded to treatment allocation by the use of coded data. As this is a feasibility trial, all analyses were treated as preliminary and exploratory, and data are reported descriptively. Continuous data are reported as median (range) or mean (SD) and categorical data as number of total (%) with 95% confidence intervals. Data are reported on an intention to treat basis.

Sample size

Since this was a feasibility study, we did not specify a specific sample size but aimed to recruit adequate participants to define the recruitment rate (primary outcome) within an approximate 10% margin of error. Based on an anticipated recruitment rate in the region of 40-50% and an anticipated number of eligible participants across the 3 centres of 130 per year, we calculated that a 1 year recruitment period would be adequate and would also allow us to fulfil the other objectives of the feasibility trial.

Patient and public involvement

Patients and families were involved in the design of the trial protocol, reviewing the patient and family facing materials and defining from the outset the outcomes to be measured. This was performed through the creation of a study specific advisory group which met prior to and regularly during the research.

Results

Trial timelines, recruitment and participants

All three centres opened to recruitment simultaneously on March 1st 2017 and were open to recruitment for 12 months until 28th February 2018. Final follow-up was completed on 31st August 2018. The trial profile is available elsewhere.[2] The primary outcome (proportion of eligible patients who were approached and recruited) was 44% (95% CI 35-51), and overall recruitment rate of those approached was 50% (95% CI 40-59). Of the 57 participants randomised, baseline characteristics were well matched (Table 1).

Table 1. Baseline characteristics of participants at randomisation

	Appendicectomy (n=28)	Non-operative treatment (n=29)

Confidential: For Review Only

**Age**	10y 7m (6y 4m – 13y 6m)	10y 3m (5y 0m– 15y 11m)
**Sex M:F (n)**	18:10	18:10*
**Duration of symptoms (hours)[%]**	32 (12-63)	34 (12-79)
**US during diagnostic work-up[$] (n (%)]**	8 (29%)	8 (28%)*
**Alvarado score[%]**	5 (3-8)	5 (3-8)
**HRQoL (mean [SD])**	0.61 (0.18)	0.57 (0.13)

Data are median (range) unless otherwise specified; US = ultrasound; HRQoL = health related quality of life measured using CHU-9D
*data for one participant who withdrew after randomisation not known
[%]note there were some missing data for duration of symptoms and Alvarado score; for duration of symptoms data are missing for 10 in appendicectomy arm and 8 (including 1 withdrawal) in non-operative treatment arm; for Alvarado score data are missing for 5 (including 1 withdrawal) in non-operative treatment arm
[$]no child had a CT scan as part of diagnostic work-up.

Confidential: For Review Only

Treatment and outcomes

Appendicectomy arm

Of 28 participants allocated to appendicectomy, one withdrew from the trial following randomisation with the remaining 27 children all having appendicectomy. Histological diagnosis of the resected appendix revealed simple acute appendicitis in 17 (63%), perforated appendicitis in eight (30%), and a normal appendix in two (7%). Median time from randomisation to hospital discharge was 63 hours (Table 2). Following discharge from hospital and during the 6 month follow-up period, complications requiring further assessment and/or treatment occurred in 8 children (30%) including hospital readmission in 3.

Non-operative treatment arm

Two of the 29 participants allocated to non-operative treatment arm withdrew following randomisation and the remaining 27 commenced non-operative treatment. One further participant withdrew from the treatment intervention requesting appendicectomy 8 hours after randomisation but permitted ongoing data collection. Nineteen of the 27 children (70%) responded to non-operative treatment and were successfully discharged from hospital. The remaining eight children underwent appendicectomy during their initial hospital admission. Reasons for appendicectomy were parental choice (withdrawal from treatment arm as previously documented) at 8 hours

following randomisation (n=1), deterioration in clinical condition (according to protocol) at range 12 to 44 hours following randomisation (n=6), and no improvement after 48 hours of non-operative treatment (in accordance with protocol, n=1). All these eight patients underwent successful appendicectomy during the initial hospital admission. Histological findings were uncomplicated acute appendicitis in four and perforated appendicitis in four. Overall median time to discharge in participants having non-operative treatment was 76 hours but was shorter (median 61 hours) in children who responded to non-operative treatment (Table 2).

Fifteen of the 29 children who underwent non-operative treatment developed at least one symptom or complication requiring assessment and/or hospital admission during the six-month follow-up. This included 7 children who developed recurrent appendicitis, of whom six underwent appendicectomy (histological findings were uncomplicated acute appendicitis [n=4] and perforated appendicitis [n=2]). The remaining participant presented with an appendix mass at the time of recurrence, which was successfully treated non-operatively and subsequently underwent interval laparoscopic appendicectomy. Two further participants were readmitted after discharge - one for assessment of abdominal pain (no further treatment required) and one for recurrent fever, treated with antibiotics but did not have appendicectomy..

Table 2. Hospital stay following randomisation

Time measure (hrs)	Total	Appendicectomy (n=27)	Non-operative treatment (n=27)	Successful non-operative treatment (n=19)	Non-operative treatment with Appendicectomy during admission (n=8)
Randomisation to decision to discharge	67 (20-196)	65 (20-196)	75 (21*-188)	59 (21-119)	115 (39 – 188)
Randomisation to actual discharge	69 (21-196)	63 (21-196)	76 (34-194)	61 (34-125)	116 (40-194)

Data are median (range); *includes 1 child who withdrew from non-operative treatment intervention but agreed for ongoing data collection

Early recovery following hospital discharge

Data relating to early post-discharge recovery were available for 26 participants, 15 in the appendicectomy arm and 11 in the non-operative treatment arm. (Figure 3). The proportion of

Confidential: For Review Only

participants reporting taking analgesia was generally lower for each day following discharge in the non-operative treatment arm than in the appendicectomy arm. Similarly, participants in the non-operative treatment arm were able to return to normal or full activities earlier than those in the appendicectomy arm; of note less than 50% of children in the appendicectomy arm reported to be able to return to full activities by 14 days. Finally, parents of participants in the non-operative treatment arm were able to return to work earlier than those in the appendicectomy arm.

Figure 3A-D. Patient outcome data reported in diary cards for trial participants in appendicectomy arm (n=16) and non-operative treatment arm (n=11). A – analgesia use; B return to normal activities; C return to full activities; D parental time off work.

A

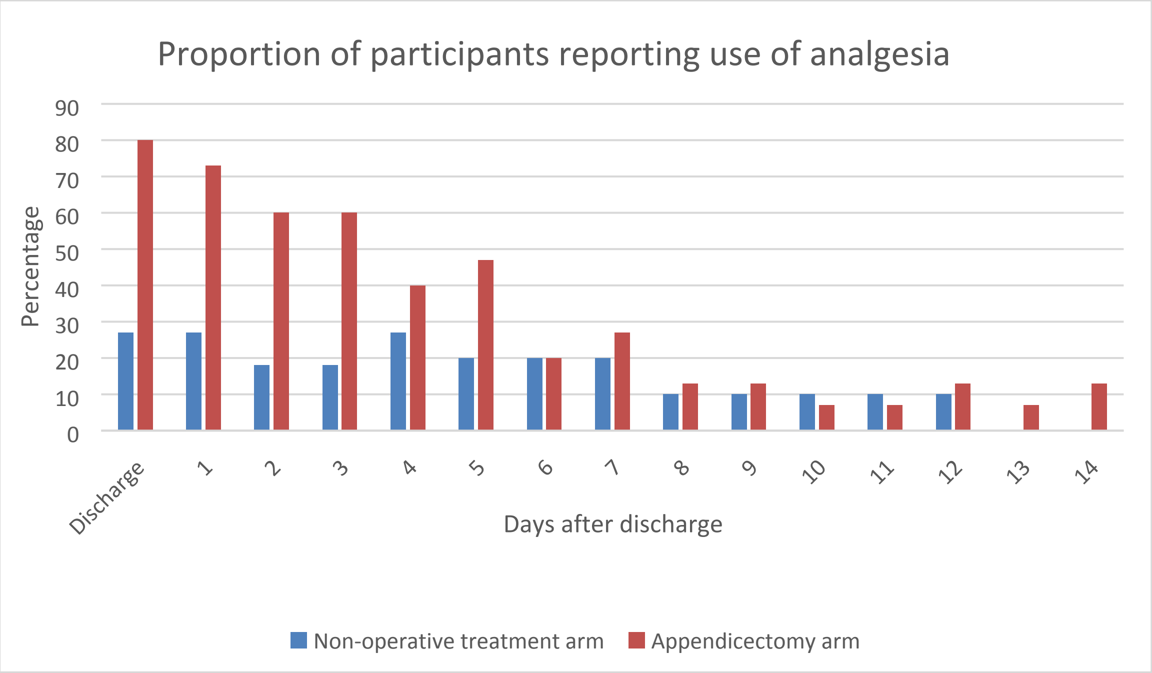

Confidential: For Review Only

B

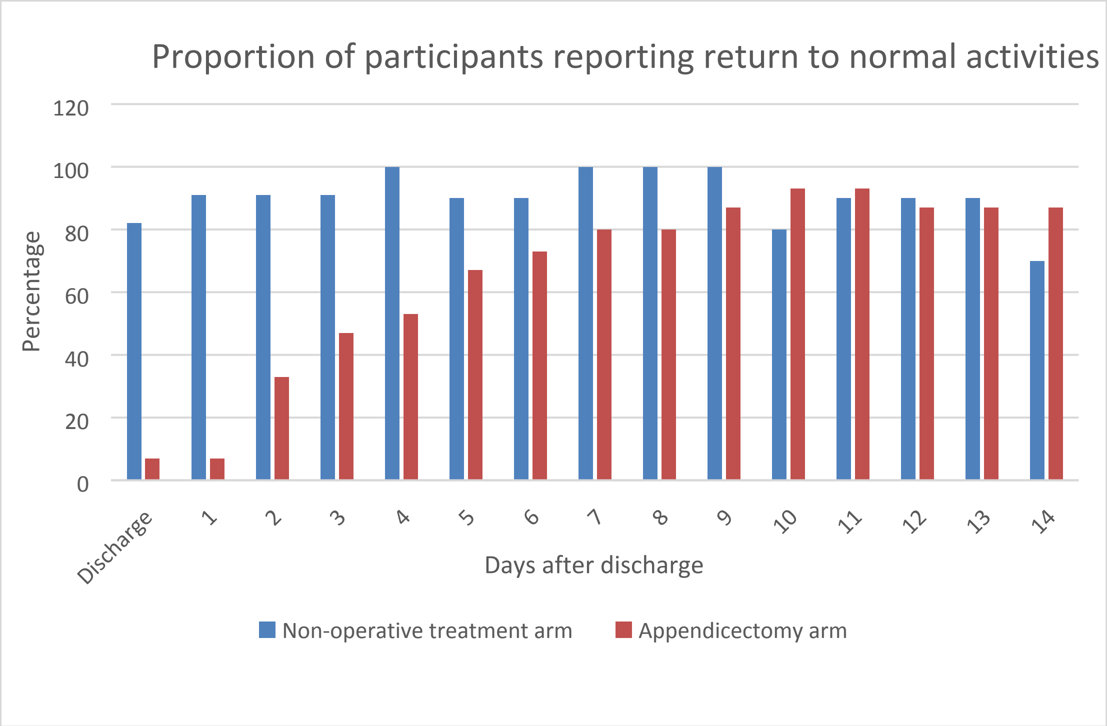
Proportion of participants reporting return to normal activities
120
100
80
60
40
20
0
Percentage
Discharge
1
2
3
4
5
6
7
8
9
10
11
12
13
14
Days after discharge
Non-operative treatment arm
Appendicectomy arm

C

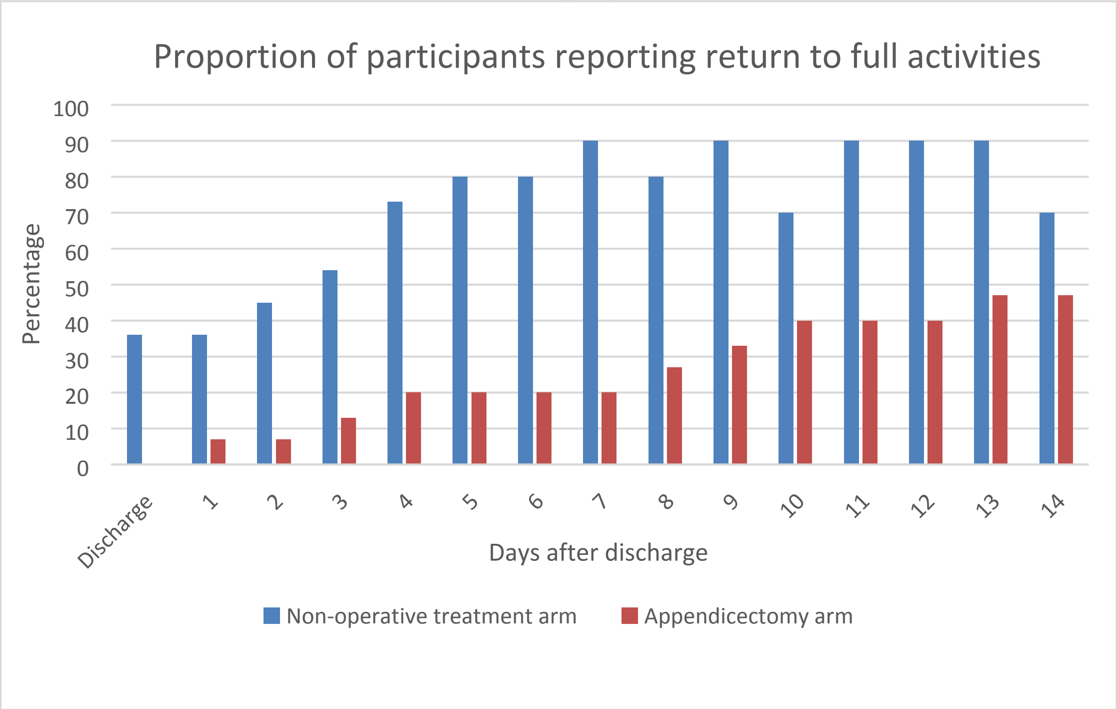
Proportion of participants reporting return to full activities
100
90
80
70
60
50
40
30
20
10
0
Percentage
Discharge
1
2
3
4
5
6
7
8
9
10
11
12
13
14
Days after discharge
Non-operative treatment arm
Appendicectomy arm

D

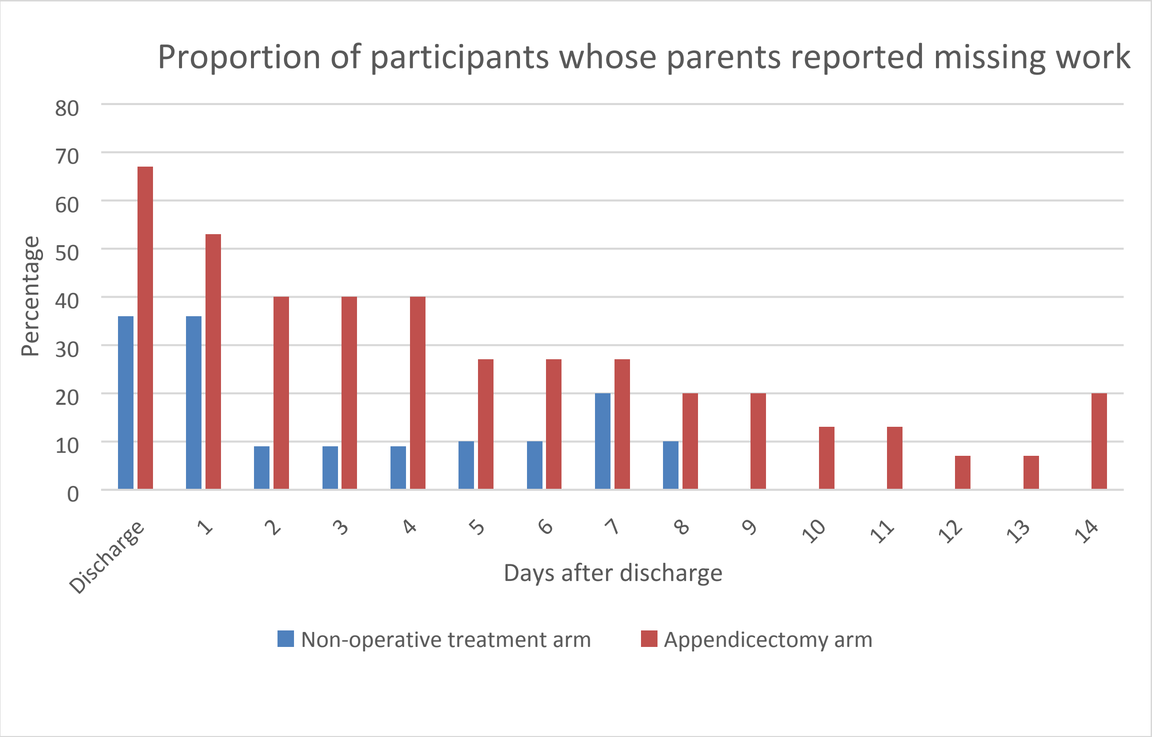

Quality of life

HRQoL assessed using the CHU-9D tool at randomisation, hospital discharge and at 4 further timepoints during the 6 month follow-up period is shown in Figure 4.

For Review Only

Figure 4 Health related quality of life (CHU-9D) during the trial.

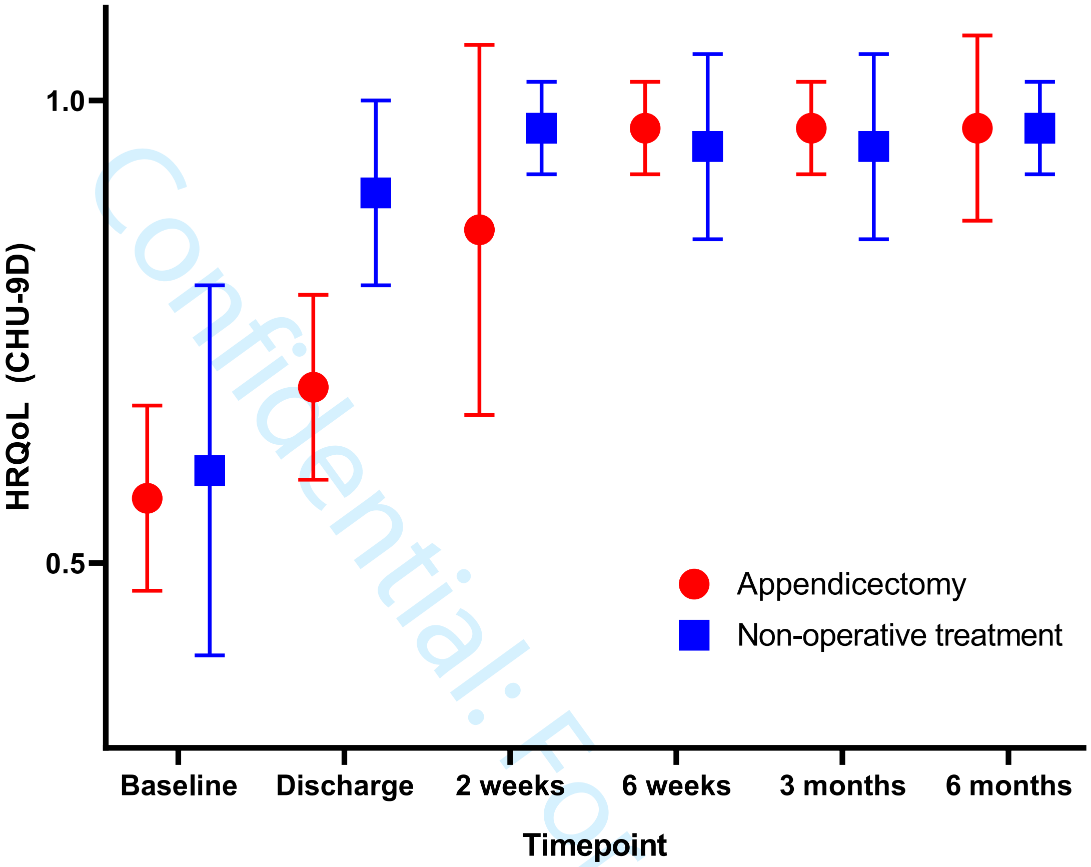

Data are mean with standard deviation
HRQoL is scored on a scale from 0 (dead) to1 (perfect health)

Discussion

In this report we describe the clinical and patient-centred outcomes of our randomised controlled feasibility trial comparing non-operative treatment with appendicectomy in children with a clinical diagnosis of uncomplicated acute appendicitis. Having previously reported that we believe performing a future randomised controlled trial comparing these two treatments is both feasible and safe[2] we now report the clinical outcomes achieved in this feasibility trial alongside outcomes that we know are important to patients and parents.[18] Our findings confirm previous data on the clinical outcomes of both appendicectomy and non-operative treatment in children with uncomplicated acute appendicitis. In addition, we report on the recovery profile of these two very different treatments by measuring patient centred outcomes during the early post discharge period. Although our sample size precludes drawing firm conclusions, our data suggest there may be

Confidential: For Review Only

differences in recovery profile of these different treatments. These data therefore highlight the importance of measuring a full range of outcomes in comparative effectiveness research including importantly those that are relevant to patients and families. If these outcomes are not formally included in trial design, they may well be overlooked.

The avoidance of surgery is a key driver for patients and parents when considering non-operative treatment for appendicitis. This encompasses a desire to avoid the trauma of surgery itself, avoidance of general anaesthesia, lack of exposure to operative/post-operative complications and the possibility of more rapid recovery from illness. When considering non-operative treatment for appendicitis these may be viewed as the potential 'benefits' over appendicectomy yet in reports to date outcomes that may be markers of these potential 'benefits' are poorly reported. Of note a recently developed core outcome set for acute uncomplicated appendicitis[18 19] includes the patient centred outcomes of 'time away from full activity', 'child's quality of life' and 'child stress/psychological distress' alongside a range of outcomes that are more traditionally of interest to clinicians/researchers. Here we report two of these alongside other measures of recovery that demonstrate impact on patients and their families of these two treatments. The pattern of recovery we have identified justifies the inclusion of these measures in future studies. Only by measuring differences in these outcomes between treatment groups will we be able to quantify fully the comparative outcomes.

Of note, since we commenced this work Minneci and colleagues have reported a large (n=1068) non-randomised study of children with uncomplicated appendicitis of whom 370 had non-operative management.[7] This is one of the only reported studies to focus on patient centred outcomes. In a propensity score matched analysis, Minneci and colleagues identified that children having non-operative management had over 4 fewer disability days in the first year following presentation with appendicitis compared to those having appendicectomy. Additionally non-operative treatment was associated with fewer patient disability days at 30 days following presentation and fewer caregiver disability days at both 30 days and 1 year following presentation. In similarity to our findings non-operative management was associated with higher quality of life scores at 30 days. Taken together these studies emphasise the importance of measing patient centred outcomes in future clinical research in this field.

The principal strengths of their study are that data were collected prospectively using predefined standard procedures in the context of a randomised control trial and that we have reported a full breadth of outcomes from a core outcome set developed for this condition. The limitations are our relatively small sample size and short duration of follow up. These were necessary due to the feasibility nature of this trial. The consequence of these limitations is that firm conclusions on the

Confidential: For Review Only

comparative outcomes between treatment arms should not be drawn from these data; rather further well designed randomised controlled trials that report a full breadth of outcomes including outcomes of importance to patients and parents should be conducted.

In conclusion, these data demonstrate the importance of measuring a full range of outcome measures in comparative effectiveness research in the field of appendicitis, rather than just outcomes of importance to clinicians or researchers. Having demonstrated that a larger multicentre trial is safe and feasible we now plan to proceed with such a trial to test the effectiveness and cost effectiveness of non-operative treatment compared to appendicectomy across a range of outcomes.

References

1. Hutchings N, Wood W, Reading I, et al. CONTRACT Study - CONservative TReatment of Appendicitis in Children (feasibility): study protocol for a randomised controlled Trial. *Trials* 2018;19(1):153. doi: 10.1186/s13063-018-2520-z
2. Hall NJ, Eaton S, Sherratt FC, et al. CONservative TReatment of Appendicitis in Children: a randomised controlled feasibility Trial (CONTRACT). *Archives of Disease in Childhood* 2021:archdischild-2020-320746. doi: 10.1136/archdischild-2020-320746
3. Xu J, Liu YC, Adams S, et al. Acute uncomplicated appendicitis study: rationale and protocol for a multicentre, prospective randomised controlled non-inferiority study to evaluate the safety and effectiveness of non-operative management in children with acute uncomplicated appendicitis. *BMJ Open* 2016;6(12):e013299. doi: 10.1136/bmjopen-2016-013299
4. Minneci PC, Deans KJ. Treatment of Uncomplicated Acute Appendicitis. *JAMA* 2015;314(13):1401-2. doi: 10.1001/jama.2015.11426
5. Hall NJ, Eaton S, Abbo O, et al. Appendectomy versus non-operative treatment for acute uncomplicated appendicitis in children: study protocol for a multicentre, open-label, non-inferiority, randomised controlled trial. *BMJ Paediatrics Open* 2017;1(1) doi: 10.1136/bmjpo-2017-000028
6. Svensson JF, Patkova B, Almstrom M, et al. Design of Studies for Antibiotic Treatment of Acute Appendicitis in Children: In Support of RCTs. *Ann Surg* 2017;266(1):e6-e7. doi: 10.1097/SLA.0000000000001291
7. Minneci PC, Hade EM, Lawrence AE, et al. Association of Nonoperative Management Using Antibiotic Therapy vs Laparoscopic Appendectomy With Treatment Success and Disability Days in Children With Uncomplicated Appendicitis. *JAMA* 2020;324(6):581. doi: 10.1001/jama.2020.10888
8. Bethell GS, Rees CM, Sutcliffe JR, et al. Management and early outcomes of children with appendicitis in the UK and Ireland during the COVID-19 pandemic: a survey of surgeons and observational study. *BMJ Paediatrics Open* 2020;4(1):e000831. doi: 10.1136/bmjpo-2020-000831
9. Kvasnovsky CL, Shi Y, Rich BS, et al. Limiting hospital resources for acute appendicitis in children: Lessons learned from the U.S. epicenter of the COVID-19 pandemic. *J Pediatr Surg* 2021;56(5):900-04. doi: 10.1016/j.jpedsurg.2020.06.024 [published Online First: 2020/07/06]
10. Hall NJ, Kapadia MZ, Eaton S, et al. Outcome reporting in randomised controlled trials and meta-analyses of appendicitis treatments in children: a systematic review. *Trials* 2015;16:275. doi: 10.1186/s13063-015-0783-1

https://mc.manuscriptcentral.com/bmjpo

Confidential: For Review Only

11. Svensson JF, Hall NJ, Eaton S, et al. A review of conservative treatment of acute appendicitis. *Eur J Pediatr Surg* 2012;22(3):185-94. doi: 10.1055/s-0032-1320014 [doi]
12. Georgiou R, Eaton S, Stanton MP, et al. Efficacy and Safety of Nonoperative Treatment for Acute Appendicitis: A Meta-analysis. *Pediatrics* 2017;139(3) doi: 10.1542/peds.2016-3003
13. Clyburne-Sherin AV, Thurairajah P, Kapadia MZ, et al. Recommendations and evidence for reporting items in pediatric clinical trial protocols and reports: two systematic reviews. *Trials* 2015;16:417. doi: 10.1186/s13063-015-0954-0 [published Online First: 2015/09/20]
14. Hall NJ, Sherratt FC, Eaton S, et al. Conservative treatment for uncomplicated appendicitis in children: the CONTRACT feasibility study, including feasibility RCT. *Health Technol Assess* 2021;25(10):1-192. doi: 10.3310/hta25100 [published Online First: 2021/02/26]
15. Eldridge SM, Chan CL, Campbell MJ, et al. CONSORT 2010 statement: extension to randomised pilot and feasibility trials. *Bmj* 2016;355:i5239. doi: 10.1136/bmj.i5239 [published Online First: 2016/10/26]
16. Collaborative RSGobotWMR. Appendicitis risk prediction models in children presenting with right iliac fossa pain (RIFT study): a prospective, multicentre validation study. *Lancet Child Adolesc Health* 2020;4(4):271-80. doi: 10.1016/S2352-4642(20)30006-7 [published Online First: 2020/03/24]
17. Tiboni S, Bhangu A, Hall NJ. Outcome of appendicectomy in children performed in paediatric surgery units compared with general surgery units. *Br J Surg* 2014;101(6):707-14. doi: 10.1002/bjs.9455 [doi]
18. Sherratt FC, Allin BSR, Kirkham JJ, et al. Core outcome set for uncomplicated acute appendicitis in children and young people. *British Journal of Surgery* 2020;107(8):1013-22. doi: 10.1002/bjs.11508
19. Sherratt FC, Eaton S, Walker E, et al. Development of a core outcome set to determine the overall treatment success of acute uncomplicated appendicitis in children: a study protocol. *BMJ Paediatr Open* 2017;1(1):e000151. doi: 10.1136/bmjpo-2017-000151

Confidential: For Review Only

Funding statement

The study was funded by the National Institute for Health Research Health Technology Assessment Program (ref: 14/192/90). The views expressed are those of the authors and not necessarily those of the NHS, the NIHR or the Department of Health. The funders had no role in developing the protocol.

Confidential: For Review Only

BMJ Paediatrics Open

Patient centred outcomes following non-operative treatment or appendicectomy for uncomplicated acute appendicitis in children

Journal:	*BMJ Paediatrics Open*
Manuscript ID	bmjpo-2022-001673.R1
Article Type:	Original research letter
Date Submitted by the Author:	23-Dec-2022
Complete List of Authors:	Hall, Nigel; University of Southampton, University Surgery Unit, Faculty of Medicine; Southampton Children's Hospital, Department of Paediatric Surgery and Urology<br>Sherratt, Frances; University of Liverpool, Department of Health Services Research, Institute of Population Health Sciences<br>Eaton, Simon; UCL Great Ormond Street Institute of Child Health Population Policy and Practice<br>Walker, Erin; Great Ormond Street Hospital For Children NHS Trust<br>Chorozoglou, Maria; University of Southampton Faculty of Medicine, Southampton Health Technology Assessment Centre<br>Beasant, Lucy; University of Bristol School of Social and Community Medicine, Centre for Child and Adolescent Health<br>Stanton, Michael; Southampton Children's Hospital, Department of Paediatric Surgery and Urology<br>Corbett, Harriet; Alder Hey Children's NHS Foundation Trust, Department of Surgery<br>Rex, Dean; St George's University Hospitals NHS Foundation Trust, Paediatric Surgery<br>Hutchings , Natalie; University of Southampton, Southampton Clinical Trials Unit, Faculty of Medicine<br>Dixon, Elizabeth; University of Southampton, Clinical Trials Unit<br>Crawley, Esther; University of Bristol School of Social and Community Medicine, Centre for Child and Adolescent Health<br>Blazeby, Jane; University of Bristol School of Social and Community Medicine, Centre for Surgical Research<br>Young, Bridget; University of Liverpool, Department of Health Services Research<br>Reading, Isabel ; University of Southampton , Primary Care and Population Sciences, Faculty of Medicine
Keywords:	Gastroenterology

I, the Submitting Author has the right to grant and does grant on behalf of all authors of the Work (as defined in the below author licence), an exclusive licence and/or a non-exclusive licence for contributions from authors who are: i) UK Crown employees; ii) where BMJ has agreed a CC-BY licence shall apply, and/or iii) in accordance with the terms applicable for US Federal Government officers or employees acting as part of their official duties; on a worldwide, perpetual, irrevocable, royalty-free basis to BMJ Publishing Group Ltd ("BMJ") its licensees and where the relevant Journal is co-owned by BMJ to the co-owners of the Journal, to publish the Work in this journal and any other BMJ products and to exploit all rights, as set out in our licence.

The Submitting Author accepts and understands that any supply made under these terms is made by BMJ to the Submitting Author unless you are acting as an employee on behalf of your employer or a postgraduate student of an affiliated institution which is paying any applicable article publishing charge ("APC") for Open Access articles. Where the Submitting Author wishes to make the Work available on an Open Access basis (and intends to pay the relevant APC), the terms of reuse of such Open Access shall be governed by a Creative Commons licence – details of these licences and which Creative Commons licence will apply to this Work are set out in our licence referred to above.

Other than as permitted in any relevant BMJ Author's Self Archiving Policies, I confirm this Work has not been accepted for publication elsewhere, is not being considered for publication elsewhere and does not duplicate material already published. I confirm all authors consent to publication of this Work and authorise the granting of this licence.

Confidential: For Review Only

Patient centred outcomes following non-operative treatment or appendicectomy for uncomplicated acute appendicitis in children

Nigel J Hall[1*], Frances C Sherratt[2], Simon Eaton[3], Erin Walker[4], Maria Chorozoglou[5], Lucy Beasant[6], Michael Stanton[7], Harriet J Corbett[8], Dean Rex[9], Natalie Hutchings[10], Elizabeth Dixon[10], Esther Crawley[6], Jane Blazeby[11], Bridget Young[2], Isabel Reading[12]

[1]Faculty of Medicine, University of Southampton, Southampton, UK
[2]Department of Health Services Research, Institute of Population Health Sciences, University of Liverpool, Liverpool UK
[3]UCL Great Ormond Street Institute of Child Health, London, UK
[4]Great Ormond Street Hospital for Children NHS Foundation Trust, London, UK
[5]Southampton Health Technology Assessment Centre, Faculty of Medicine, University of Southampton, Southampton, UK
[6]Centre for Child and Adolescent Health, School of Social and Community Medicine, University of Bristol
[7]Department of Paediatric Surgery and Urology, Southampton Children's Hospital, University Hospital Southampton NHS Foundation Trust, UK
[8]Department of Paediatric Surgery, Alder Hey Children's NHS foundation Trust, East Prescott Road, Liverpool
[9]Department of Paediatric Surgery, St George's Hospital NHS foundation Trust, Tooting, London
[10]Southampton Clinical Trials Unit, University of Southampton, Southampton, UK
[11]Centre for Surgical Research, and the NIHR Bristol and Weston Biomedical Research Centre, Population Health Sciences, Bristol Medical School, University of Bristol and University Hospitals Bristol NHS Foundation Trust
[12]Primary Care and Population Sciences, Faculty of Medicine, University of Southampton, Southampton, UK

*Corresponding author

The authors have no competing interests to declare

Word count: Abstract 99; Main text 598

Funding statement

The study was funded by the National Institute for Health Research Health Technology Assessment Program (ref: 14/192/90). The views expressed are those of the authors and not necessarily those of the NHS, the NIHR or the Department of Health. The funders had no role in developing the protocol.

Confidential: For Review Only

Abstract

Whilst non-operative treatment has emerged as an alternative to surgery for the treatment of uncomplicated acute appendicitis in children, comparative patient centred outcomes are not well documented. We investigated these in a feasibility randomised trial. Of 57 randomised participants, data were available for 26. Compared to appendicectomy, children allocated to non-operative treatment reported higher short term quality of life scores, shorter duration of requiring analgesia, more rapid return to normal activities and shorter parental absence from work. These preliminary data suggest differences exist in recovery profile and quality of life between these treatments that are important to measure in a larger RCT.

Trial registration ISRCTN15830435.

Confidential: For Review Only

Treatment of appendicitis in children has traditionally been by appendicectomy. Over recent years there has been increasing interest in the use of non-operative treatment as an alternative to appendicectomy[1 2] but comparative outcomes of these two different treatment modalities are poorly understood. Previous reports have tended to focus on outcomes most relevant to surgeons such as surgical complications and other treatments required rather than considering a full range of outcomes that we know are important to patients and families.[3] Here we report a number of patient centred outcomes including health related quality of life (HRQoL) in data arising from a feasibility randomised controlled trial.

A feasibility RCT comparing appendicectomy and non-operative treatment in children with suspected uncomplicated acute appendicitis was undertaken in three United Kingdom centres over a 12 month period beginning March 2017. Full details of the methodology, key clinical findings and feasibility findings of the trial have been previously reported.[4] To understand patient centred markers of recovery we used parent completed diary cards following hospital discharge to record duration of analgesia use, time taken to return to normal and full activities and duration of parental absence from work. We used the CHU-9D tool[5] to measure HRQoL during the trial period. Since this was a feasibility trial, we report descriptive data without formal statistical analyses. Data are reported on an intention to treat basis as per trial allocation.

Patients and families were involved in the design of the trial, development of patient and family facing materials and defining outcomes to be measured.

A total of 57 participants were enrolled in the trial of whom 28 were allocated to appendicectomy and 29 to non-operative treatment. Data relating to early post-discharge recovery were available for 26 participants - 15 in the appendicectomy arm and 11 in the non-operative treatment arm. The proportion of participants taking analgesia was generally lower for each day following discharge in the non-operative treatment arm than the appendicectomy arm. Following non-operative treatment participants were able to return to normal or full activities earlier than those in the appendicectomy arm. Fewer than half of participants in the appendicectomy reported being able to participate in full activities by two weeks following discharge. Parents of participants in the non-operative treatment arm were able to return to work earlier than those in the appendicectomy arm. Health related quality of life assessed using the CHU-9D tool at enrolment, hospital discharge and further time points during the six month follow up period is shown in Figure 1.

Whilst we acknowledge a relatively small sample size and incomplete data, our observation of an apparent difference in early post-discharge recovery profile between participants allocated to appendicectomy or non-operative treatment is an important finding with relevance for both clinical practice and future research. Avoidance of general anaesthesia, trauma of surgery and lack of exposure to operative/ post-operative complications are viewed as potential benefits of non-operative treatment over surgery by patients and parents. Our data suggest there may be additional benefits to non-operative treatment over surgery in terms of more rapid return to baseline activity status and societal benefits with fewer days of parental work absence. Patients and parents may wish to consider these as well as more direct clinical outcomes when considering choice of treatment. Furthermore, there appear to be short term differences in HRQoL between treatment arms which may clinically meaningful and will inform design of future cost-utility analyses.

Overall our data emphasise the importance of measuring a full range of outcomes including these patient centred outcomes in future research and provide greater insights into short term differences in outcomes between treatment arms.

Confidential: For Review Only

Table 1: Profile of post-discharge recovery in each treatment arm

Day following discharge	Analgesia used		Able to do normal activities		Able to do full activities		Parental work absence	
	NOT	APP	NOT	APP	NOT	APP	NOT	APP
0	27	80	82	7	36	0	36	67
1	27	73	91	7	36	7	36	53
2	18	60	91	33	45	7	9	40
3	18	60	91	47	54	13	9	40
4	27	40	100	53	73	20	9	40
5	20	47	90	67	80	20	10	27
6	20	20	90	73	80	20	10	27
7	20	27	100	80	90	20	20	27
8	10	13	100	80	80	27	10	20
9	10	13	100	87	90	33	0	20
10	10	7	80	93	70	40	0	13
11	10	7	90	93	90	40	0	13
12	10	13	90	87	90	40	0	7
13	0	7	90	87	90	47	0	7
14	0	13	70	87	70	47	0	20

Data are percentage of participants reporting data on each day.

NOT – non-operative treatment arm; APP – appendicectomy arm

Confidential: For Review Only

Figure 1: Health related quality of life (CHU-9D) during the trial.

Data are mean with standard deviation
HRQoL is scored on a scale from 0 (dead) to1 (perfect health)

Confidential: For Review Only

References

1. Georgiou R, Eaton S, Stanton MP, et al. Efficacy and Safety of Nonoperative Treatment for Acute Appendicitis: A Meta-analysis. *Pediatrics* 2017;139(3) doi: 10.1542/peds.2016-3003
2. Minneci PC, Hade EM, Lawrence AE, et al. Association of Nonoperative Management Using Antibiotic Therapy vs Laparoscopic Appendectomy With Treatment Success and Disability Days in Children With Uncomplicated Appendicitis. *JAMA* 2020;324(6):581. doi: 10.1001/jama.2020.10888
3. Sherratt FC, Allin BSR, Kirkham JJ, et al. Core outcome set for uncomplicated acute appendicitis in children and young people. *Br J Surg* 2020 doi: 10.1002/bjs.11508 [published Online First: 2020/03/18]
4. Hall NJ, Eaton S, Sherratt FC, et al. CONservative TReatment of Appendicitis in Children: a randomised controlled feasibility Trial (CONTRACT). *Archives of Disease in Childhood* 2021:archdischild-2020-320746. doi: 10.1136/archdischild-2020-320746
5. Stevens KJ. Assessing the performance of a new generic measure of health related quality of life for children and refining it for use in health state valuation. *Applied Health Economics and Health Policy* 2011;9:157-69.

Confidential: For Review Only

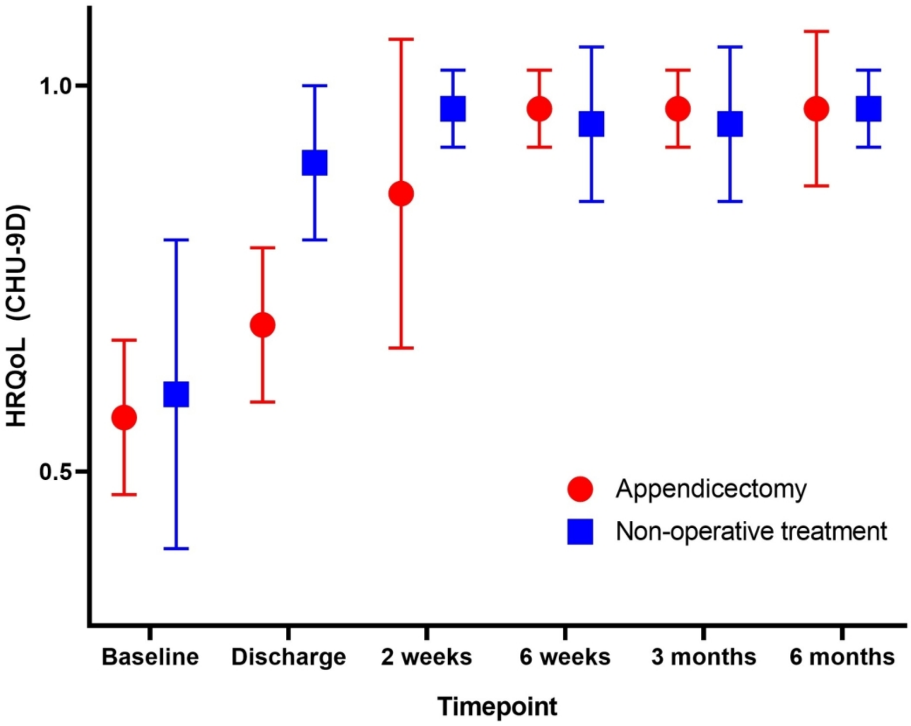

Figure 1: Health related quality of life (CHU-9D) during the trial.

Data are mean with standard deviation
HRQoL is scored on a scale from 0 (dead) to1 (perfect health)

158x123mm (300 x 300 DPI)

BMJ Paediatrics Open

Patient centred outcomes following non-operative treatment or appendicectomy for uncomplicated acute appendicitis in children

Journal:	*BMJ Paediatrics Open*
Manuscript ID	bmjpo-2022-001673.R2
Article Type:	Original research letter
Date Submitted by the Author:	11-Jan-2023
Complete List of Authors:	Hall, Nigel; University of Southampton, University Surgery Unit, Faculty of Medicine; Southampton Children's Hospital, Department of Paediatric Surgery and Urology<br>Sherratt, Frances; University of Liverpool, Department of Health Services Research, Institute of Population Health Sciences<br>Eaton, Simon; UCL Great Ormond Street Institute of Child Health Population Policy and Practice<br>Walker, Erin; Great Ormond Street Hospital For Children NHS Trust<br>Chorozoglou, Maria; University of Southampton Faculty of Medicine, Southampton Health Technology Assessment Centre<br>Beasant, Lucy; University of Bristol School of Social and Community Medicine, Centre for Child and Adolescent Health<br>Stanton, Michael; Southampton Children's Hospital, Department of Paediatric Surgery and Urology<br>Corbett, Harriet; Alder Hey Children's NHS Foundation Trust, Department of Surgery<br>Rex, Dean; St George's University Hospitals NHS Foundation Trust, Paediatric Surgery<br>Hutchings , Natalie; University of Southampton, Southampton Clinical Trials Unit, Faculty of Medicine<br>Dixon, Elizabeth; University of Southampton, Clinical Trials Unit<br>Crawley, Esther; University of Bristol School of Social and Community Medicine, Centre for Child and Adolescent Health<br>Blazeby, Jane; University of Bristol School of Social and Community Medicine, Centre for Surgical Research<br>Young, Bridget; University of Liverpool, Department of Health Services Research<br>Reading, Isabel ; University of Southampton , Primary Care and Population Sciences, Faculty of Medicine
Keywords:	Gastroenterology

Confidential: For Review Only

I, the Submitting Author has the right to grant and does grant on behalf of all authors of the Work (as defined in the below author licence), an exclusive licence and/or a non-exclusive licence for contributions from authors who are: i) UK Crown employees; ii) where BMJ has agreed a CC-BY licence shall apply, and/or iii) in accordance with the terms applicable for US Federal Government officers or employees acting as part of their official duties; on a worldwide, perpetual, irrevocable, royalty-free basis to BMJ Publishing Group Ltd ("BMJ") its licensees and where the relevant Journal is co-owned by BMJ to the co-owners of the Journal, to publish the Work in this journal and any other BMJ products and to exploit all rights, as set out in our licence.

The Submitting Author accepts and understands that any supply made under these terms is made by BMJ to the Submitting Author unless you are acting as an employee on behalf of your employer or a postgraduate student of an affiliated institution which is paying any applicable article publishing charge ("APC") for Open Access articles. Where the Submitting Author wishes to make the Work available on an Open Access basis (and intends to pay the relevant APC), the terms of reuse of such Open Access shall be governed by a Creative Commons licence – details of these licences and which Creative Commons licence will apply to this Work are set out in our licence referred to above.

Other than as permitted in any relevant BMJ Author's Self Archiving Policies, I confirm this Work has not been accepted for publication elsewhere, is not being considered for publication elsewhere and does not duplicate material already published. I confirm all authors consent to publication of this Work and authorise the granting of this licence.

Patient centred outcomes following non-operative treatment or appendicectomy for uncomplicated acute appendicitis in children

Nigel J Hall[1*], Frances C Sherratt[2], Simon Eaton[3], Erin Walker[4], Maria Chorozoglou[5], Lucy Beasant[6], Michael Stanton[7], Harriet J Corbett[8], Dean Rex[9], Natalie Hutchings[10], Elizabeth Dixon[10], Esther Crawley[6], Jane Blazeby[11], Bridget Young[2], Isabel Reading[12]

[1]Faculty of Medicine, University of Southampton, Southampton, UK
[2]Department of Health Services Research, Institute of Population Health Sciences, University of Liverpool, Liverpool UK
[3]UCL Great Ormond Street Institute of Child Health, London, UK
[4]Great Ormond Street Hospital for Children NHS Foundation Trust, London, UK
[5]Southampton Health Technology Assessment Centre, Faculty of Medicine, University of Southampton, Southampton, UK
[6]Centre for Child and Adolescent Health, School of Social and Community Medicine, University of Bristol
[7]Department of Paediatric Surgery and Urology, Southampton Children's Hospital, University Hospital Southampton NHS Foundation Trust, UK
[8]Department of Paediatric Surgery, Alder Hey Children's NHS foundation Trust, East Prescott Road, Liverpool
[9]Department of Paediatric Surgery, St George's Hospital NHS foundation Trust, Tooting, London
[10]Southampton Clinical Trials Unit, University of Southampton, Southampton, UK
[11]Centre for Surgical Research, and the NIHR Bristol and Weston Biomedical Research Centre, Population Health Sciences, Bristol Medical School, University of Bristol and University Hospitals Bristol NHS Foundation Trust
[12]Primary Care and Population Sciences, Faculty of Medicine, University of Southampton, Southampton, UK

*Corresponding author

The authors have no competing interests to declare

Word count: Abstract 99; Main text 598

Funding statement

The study was funded by the National Institute for Health Research Health Technology Assessment Program (ref: 14/192/90). The views expressed are those of the authors and not necessarily those of the NHS, the NIHR or the Department of Health. The funders had no role in developing the protocol.

Confidential: For Review Only

Abstract

Whilst non-operative treatment has emerged as an alternative to surgery for the treatment of uncomplicated acute appendicitis in children, comparative patient centred outcomes are not well documented. We investigated these in a feasibility randomised trial. Of 57 randomised participants, data were available for 26. Compared to appendicectomy, children allocated to non-operative treatment reported higher short term quality of life scores, shorter duration of requiring analgesia, more rapid return to normal activities and shorter parental absence from work. These preliminary data suggest differences exist in recovery profile and quality of life between these treatments that are important to measure in a larger RCT.

Trial registration ISRCTN15830435.

Confidential: For Review Only

Treatment of appendicitis in children has traditionally been by appendicectomy. Over recent years there has been increasing interest in the use of non-operative treatment as an alternative to appendicectomy[1 2] but comparative outcomes of these two different treatment modalities are poorly understood. Previous reports have tended to focus on outcomes most relevant to surgeons such as surgical complications and other treatments required rather than considering a full range of outcomes that we know are important to patients and families.[3] Here we report a number of patient centred outcomes including health related quality of life (HRQoL) in data arising from a feasibility randomised controlled trial.

A feasibility RCT comparing appendicectomy and non-operative treatment in children with suspected uncomplicated acute appendicitis was undertaken in three United Kingdom centres over a 12 month period beginning March 2017. Full details of the methodology, key clinical findings and feasibility findings of the trial have been previously reported.[4] To understand patient centred markers of recovery we used parent completed diary cards following hospital discharge to record duration of analgesia use, time taken to return to normal and full activities and duration of parental absence from work. We used the CHU-9D tool[5] to measure HRQoL during the trial period. Since this was a feasibility trial, we report descriptive data without formal statistical analyses. Data are reported on an intention to treat basis as per trial allocation.

Patients and families were involved in the design of the trial, development of patient and family facing materials and defining outcomes to be measured.

A total of 57 participants were enrolled in the trial of whom 28 were allocated to appendicectomy and 29 to non-operative treatment. Data relating to early post-discharge recovery were available for 26 participants - 15 in the appendicectomy arm and 11 in the non-operative treatment arm. The proportion of participants taking analgesia was generally lower for each day following discharge in the non-operative treatment arm than the appendicectomy arm. Following non-operative treatment participants were able to return to normal or full activities earlier than those in the appendicectomy arm. Fewer than half of participants in the appendicectomy reported being able to participate in full activities by two weeks following discharge. Parents of participants in the non-operative treatment arm were able to return to work earlier than those in the appendicectomy arm. Health related quality of life assessed using the CHU-9D tool at enrolment, hospital discharge and further time points during the six month follow up period is shown in Figure 1.

Whilst we acknowledge a relatively small sample size and incomplete data, our observation of an apparent difference in early post-discharge recovery profile between participants allocated to appendicectomy or non-operative treatment is an important finding with relevance for both clinical practice and future research. Avoidance of general anaesthesia, trauma of surgery and lack of exposure to operative/ post-operative complications are viewed as potential benefits of non-operative treatment over surgery by patients and parents. Our data suggest there may be additional benefits to non-operative treatment over surgery in terms of more rapid return to baseline activity status and societal benefits with fewer days of parental work absence. Patients and parents may wish to consider these as well as more direct clinical outcomes when considering choice of treatment. Furthermore, there appear to be short term differences in HRQoL between treatment arms which may clinically meaningful and will inform design of future cost-utility analyses.

Overall our data emphasise the importance of measuring a full range of outcomes including these patient centred outcomes in future research and provide greater insights into short term differences in outcomes between treatment arms.

Confidential: For Review Only

Table 1: Profile of post-discharge recovery in each treatment arm

Day following discharge	Analgesia used		Able to do normal activities		Able to do full activities		Parental work absence	
	NOT	APP	NOT	APP	NOT	APP	NOT	APP
0	27	80	82	7	36	0	36	67
1	27	73	91	7	36	7	36	53
2	18	60	91	33	45	7	9	40
3	18	60	91	47	54	13	9	40
4	27	40	100	53	73	20	9	40
5	20	47	90	67	80	20	10	27
6	20	20	90	73	80	20	10	27
7	20	27	100	80	90	20	20	27
8	10	13	100	80	80	27	10	20
9	10	13	100	87	90	33	0	20
10	10	7	80	93	70	40	0	13
11	10	7	90	93	90	40	0	13
12	10	13	90	87	90	40	0	7
13	0	7	90	87	90	47	0	7
14	0	13	70	87	70	47	0	20

Data are percentage of participants reporting data on each day. For all outcomes, n=15 for APP arm and n=11 for NOT arm for days 0-4 and n=10 for days 5-14.

NOT – non-operative treatment arm; APP – appendicectomy arm

Confidential: For Review Only

Figure 1: Health related quality of life (CHU-9D) during the trial.

Data are mean with standard deviation
HRQoL is scored on a scale from 0 (dead) to1 (perfect health)

Confidential: For Review Only

References

1. Georgiou R, Eaton S, Stanton MP, et al. Efficacy and Safety of Nonoperative Treatment for Acute Appendicitis: A Meta-analysis. *Pediatrics* 2017;139(3) doi: 10.1542/peds.2016-3003
2. Minneci PC, Hade EM, Lawrence AE, et al. Association of Nonoperative Management Using Antibiotic Therapy vs Laparoscopic Appendectomy With Treatment Success and Disability Days in Children With Uncomplicated Appendicitis. *JAMA* 2020;324(6):581. doi: 10.1001/jama.2020.10888
3. Sherratt FC, Allin BSR, Kirkham JJ, et al. Core outcome set for uncomplicated acute appendicitis in children and young people. *Br J Surg* 2020 doi: 10.1002/bjs.11508 [published Online First: 2020/03/18]
4. Hall NJ, Eaton S, Sherratt FC, et al. CONservative TReatment of Appendicitis in Children: a randomised controlled feasibility Trial (CONTRACT). *Archives of Disease in Childhood* 2021:archdischild-2020-320746. doi: 10.1136/archdischild-2020-320746
5. Stevens KJ. Assessing the performance of a new generic measure of health related quality of life for children and refining it for use in health state valuation. *Applied Health Economics and Health Policy* 2011;9:157-69.

Confidential: For Review Only

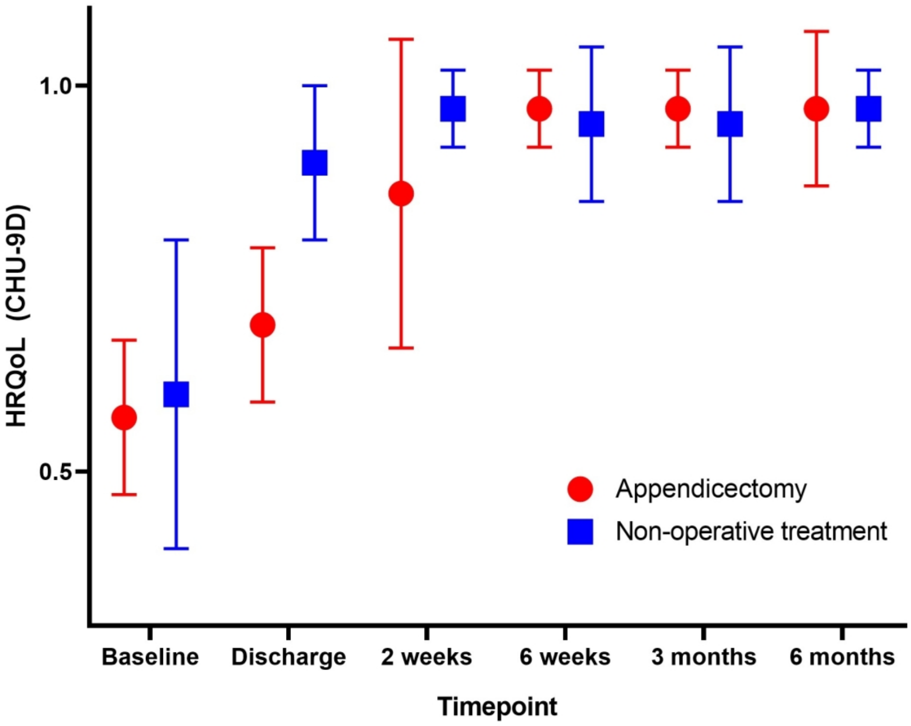

Figure 1: Health related quality of life (CHU-9D) during the trial.

Data are mean with standard deviation
HRQoL is scored on a scale from 0 (dead) to1 (perfect health)

158x123mm (330 x 330 DPI)
